# *Rickettsia parkeri* Rickettsiosis, Argentina

**DOI:** 10.3201/eid1707.101857

**Published:** 2011-07

**Authors:** Yamila Romer, Alfredo C. Seijo, Favio Crudo, William L. Nicholson, Andrea Varela-Stokes, R. Ryan Lash, Christopher D. Paddock

**Affiliations:** Author affiliations: Hospital F.J. Muñiz, Buenos Aires, Argentina (Y. Romer, A.C. Seijo, F. Crudo);; Centers for Disease Control and Prevention, Atlanta, Georgia, USA (W.L. Nicholson, C.D. Paddock);; Mississippi State University, Mississippi State, Mississippi, USA (A. Varela-Stokes);; The University of Georgia, Athens, Georgia, USA (R.R. Lash)

## Abstract

*Rickettsia parkeri*, a recently identified cause of spotted fever rickettsiosis in the United States, has been found in *Amblyomma* *triste* ticks in several countries of South America, including Argentina, where it is believed to cause disease in humans. We describe the clinical and epidemiologic characteristics of 2 patients in Argentina with confirmed *R. parkeri* infection and 7 additional patients with suspected *R. parkeri* rickettsiosis identified at 1 hospital during 2004–2009. The frequency and character of clinical signs and symptoms among these 9 patients closely resembled those described for patients in the United States (presence of an inoculation eschar, maculopapular rash often associated with pustules or vesicles, infrequent gastrointestinal manifestations, and relatively benign clinical course). Many *R. parkeri* infections in South America are likely to be misdiagnosed as other infectious diseases, including Rocky Mountain spotted fever, dengue, or leptospirosis.

*Rickettsia parkeri*, a tick-borne bacterium discovered in 1937, was considered nonpathogenic until 2004. Since 2004, >25 cases of *R. parkeri* rickettsiosis have been reported in persons living within the recognized range of the tick vector, *Amblyomma maculatum*, in the United States (*1*–*4*; Centers for Disease Control and Prevention, unpub. data). The clinical features of this newly recognized disease appear less severe than those produced by *R. rickettsii* bacteria, the agent of Rocky Mountain spotted fever (RMSF). For many years, investigators in several countries of South America, including Argentina, Brazil, and Uruguay, have recognized eschar-associated infections that clinically resemble *R. parkeri* rickettsiosis (*5**–**7*). These reports, and the discoveries of *R. parkeri* in *A. triste* ticks collected from these same countries, suggest that human infections with *R. parkeri* also occur in South America (*8**–**10*); to our knowledge, no confirmed cases of disease caused by this *Rickettsia* species have been reported from this continent.

The Paraná Delta, situated in the provinces of Buenos Aires and Entre Ríos in Argentina, represents the terminus of the Paraná River as it approaches and drains into the Uruguay River and subsequently into the Río de la Plata. This alluvial ecosystem, where braided river branches create a network of islands and wetlands, covers ≈14,000 km^2^ (5,405 mi^2^) and extends for ≈320 km (200 mi). This region also contains abundant populations of *A. triste* ticks (*10*). The Paraná Delta has always been a major agricultural and farming region. Recently, this area has become increasingly developed; roads have been built to allow greater access for tourism and recreational activities by many of the ≈14 million inhabitants of nearby Buenos Aires. In 2005, an eschar-associated febrile infection was diagnosed in a male beekeeper from the Paraná Delta; the infection was later confirmed as a spotted fever group (SFG) rickettsiosis by serology and immunohistochemistry (*5*). He had been bitten by a tick not far from several sites where *R. parkeri* was subsequently detected in *A. triste* ticks (*10*). We report confirmed cases of *R. parkeri* rickettsiosis in 2 patients in Argentina and describe additional suspected cases of this disease, or similar infections, in patients from the provinces of Buenos Aires, Chaco, and Entre Ríos.

## Materials and Methods

Patients were identified after referral to the Zoonosis Service of Hospital F.J. Muñiz in Buenos Aires Province, Argentina. In each case, a rickettsial disease was considered from specific clinical signs and symptoms, including fever, rash, and an eschar, accompanying a history of recent tick bite. Serum and skin biopsy specimens were collected from these patients and evaluated by various assays to confirm infection with an SFG *Rickettsia* species. Serum samples were tested for immunoglobulin (Ig) G reactive to antigens of *R. parkeri* and *R. rickettsii* by using indirect immunofluorescence antibody assays, as described (*1**,**3*). Reciprocal antibody titers >64, or a 4-fold rise in titer, to either antigen were considered evidence of infection with an SFG *Rickettsia* species. When available, skin biopsy specimens were tested by using an immunoalkaline phosphatase technique to detect SFG rickettsiae in formalin-fixed, paraffin-embedded tissues, as described (*1**,**11*), or by use of PCR.

For molecular evaluations, DNA was extracted from eschar biopsy specimens by using a QIAamp DNA Mini Kit (QIAGEN, Valencia, CA, USA). A segment of the rickettsial outer membrane protein B gene (*ompB*) was amplified by using primers 120–2,788 and 120–3,599 (*12*) in a 50-µL reaction mixture containing 5 µL of DNA template. A segment of the citrate synthase gene (*gltA*) was also amplified by using primers CS78 and CS323 (*13*) in a 40-µL reaction mixture containing 8 µL of DNA template. Amplified gene segments, excluding primers, were compared with sequences in the GenBank database by using the Basic Local Alignment Search Tool (National Center for Biotechnology Information, www.ncbi.nlm.nih.gov). Gene sequences were aligned by using ClustalX software (*14*).

A suspected case was defined as a clinically and epidemiologically compatible illness, with *>*1 supportive serologic or immunohistochemical test results through use of group-specific assays for SFG *Rickettsia* spp (*11*). A confirmed case of *R. parkeri* rickettsiosis was defined by PCR amplification of gene sequences specifically matching that of *R. parkeri*.

## Results

**Figure 1:**
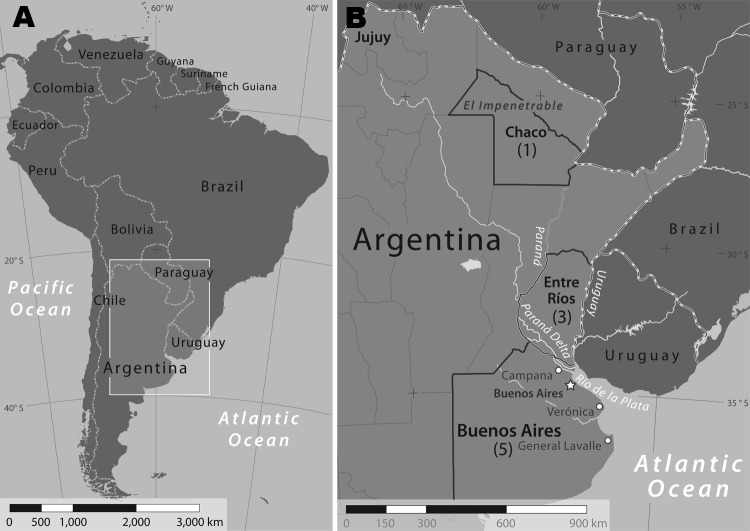
Confirmed and suspected cases of *Rickettsia parkeri* rickettsiosis, Argentina*.* The box (A) enlarged in panel (B) shows the extent of the area in which Argentinean provinces, representing patient exposure locations to ticks, are labeled and highlighted. A previous study (*10*) identified ticks collected from the Paraná Delta near the city of Campana. Numbers of suspected and confirmed cases of *R. parkeri* rickettsiosis, by province during 2004–2009, are shown in parentheses. The national capital city of Buenos Aires continues to experience rapid population growth into adjacent lands in and near the Paraná Delta. Rocky Mountain spotted fever, a more severe tick-borne rickettsiosis, has been described in the province of Jujuy in the northwestern corner of Argentina (*15*,*16*).

During 2004–2009, nine patients in Argentina with an SFG rickettsiosis were identified at Hospital F.J. Muñiz. Three were women. Median age of patients was 53 years (range 38–76 years). All patients reported tick bites that occurred during August–January and preceded fever onset by a median of 6 days (range 4–15 days). Exposures to ticks were associated with recreational activities for 7 patients and outdoor labor for 2. Six patients sustained tick bites in the Paraná Delta; 2 other patients were bitten in rural areas of the province of Buenos Aires near the towns of Verónica and General Lavalle; and 1 was bitten in a rural area of the province of Chaco, known as “El Impenetrable” (Figure 1, panel B).

**Figure 2:**
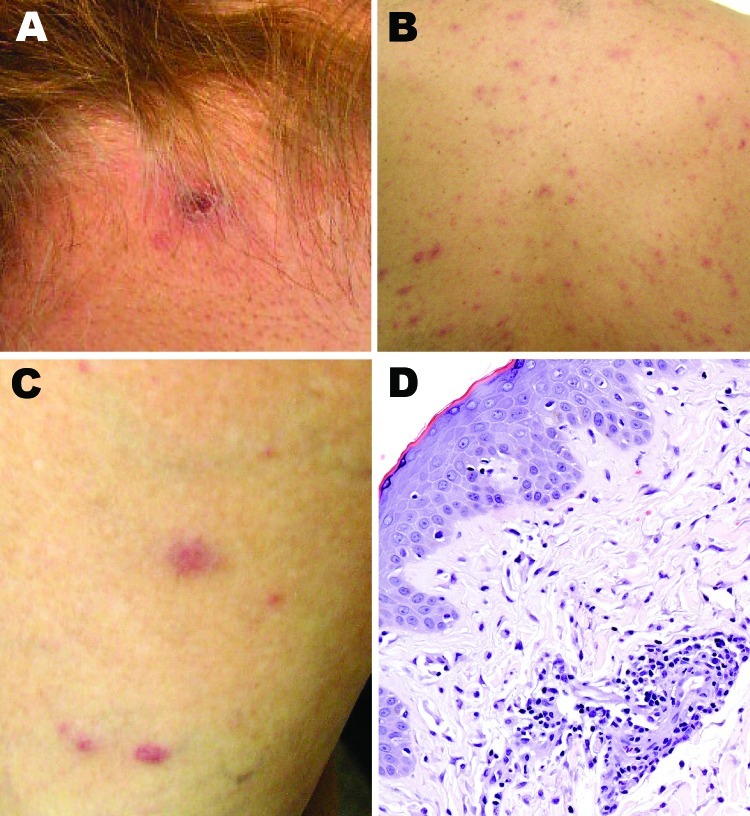
Cutaneous lesions of patients with suspected and confirmed *Rickettsia parkeri* rickettsiosis in Argentina. A) Eschar at the nape of the neck at the site of recent tick bite. B, C) Papulovesicular rash involving the back and lower extremities. D) Histopathologic appearance of a papule biopsy specimen, showing perivascular mononuclear inflammatory cell infiltrates and edema of the adjacent superficial dermis and an intact epidermis (hematoxylin and eosin stain; original magnification ×100).

A painless inoculation eschar, ranging from 1 cm to 1.5 cm, developed in 8 patients at the site of the tick bite (Table; Figure 2). These lesions were located in the following regions: head (3 patients); back (2); and leg, hand, and abdomen (1 each). Multiple eschars were not identified on any patient. Nonpruritic rashes developed in all patients and involved predominantly the trunk and extremities, represented by maculopapules on 8 patients, papulovesicles on 5 patients, and petechiae on 2 patients (Figure 2, panels B, C). Other commonly reported manifestations included headache and myalgias in 8 and 6 patients, respectively. Infrequently reported findings included arthralgias (3 patients); sore throat (2); and diarrhea, photophobia, and bilaterally injected conjunctivae (1 each). No patients required hospitalization, and all recovered rapidly after oral therapy with doxycycline.

Laboratory identification of cases included serology for 7 patients, immunohistochemistry for 1, and PCR for 2. Seroconversion, defined as a 4-fold increase in titer, was identified for 5 patients submitting paired serum samples, and 1 serum sample was positive in 2 patients; IgG titers to *R. parkeri* or *R. rickettsii* antigens ranged from 64 to 2,048. SFG *Rickettsia* spp. antigens were detected in formalin-fixed, paraffin-embedded sections of eschar and papule biopsy specimens that showed histopathologic features (Figure 2, panel D) compatible with those described for *R. parkeri* rickettsiosis (*3**,**11*). Infection with *R. parkeri* was confirmed specifically by molecular analyses of eschar biopsy specimens from 2 patients whose histories are provided below. Amplicons of the expected sizes were obtained from both specimens by using the primers for the rickettsial *ompB* and *gltA* genes. Sequence analyses of amplified *ompB* segments showed 100% identity with only the corresponding sequence of *R. parkeri* (GenBank accession no. AF123717). The next closest similarities were 711/714 (99%) with *Rickettsia* sp. BJ-90 (accession no. AY331393), 711/714 (99%) with *R. sibirica* (accession no. AF123722), and 710/714 with *R. africae* (accession no. CP001612). Sequencing of 332 bp of the *gltA* gene showed 100% identity with *R. parkeri* (accession no. U59732) and with *Rickettsia* sp. strain S (accession no. U59735), *R. sibirica* (accession no. U59734), and *Rickettsia* sp. BJ-90 (accession no. AF178035). Complete identity was also seen with a smaller (309-bp) overlapping segment of the *gltA* gene of *Rickettsia* sp. Atlantic rainforest (accession no. GQ855235), *Rickettsia* sp. NOD (accession no. EU567177), and *Rickettsia* sp. COOPERI (accession no. AY362704).

## Case Histories

Patient 1, a 48-year-old man, was referred to F.J. Muñiz Hospital in November 2008 with recurring fever (temperatures to 40ºC) associated with headache, neck pain, and rash. Six days before onset of these symptoms, he removed a tick attached to his abdomen, ≈24 hours after a fishing expedition in the Paraná Delta in the province of Entre Ríos (Figure 1, panel B). A painless eschar developed at the bite site 6 days after the tick was removed. Physical examination indicated a febrile patient in otherwise good condition with a generalized, nonpruritic, maculopapular, and papulovesicular rash that involved predominantly his trunk and upper and lower limbs; his face, palms, or soles were not affected. His serum alanine aminotransferase level was 43 U/L (reference <31 U/L). All other hematologic and biochemistry measurements were within reference ranges. A serum sample and eschar and papule biopsy specimens were obtained, and the patient was treated empirically with doxycycline 200 mg/day for 6 days.

Patient 2, a previously healthy 55-year-old-man, was referred to F.J. Muñiz Hospital in January 2009 with fever (temperature 38.5°C), rash, headache, photophobia, conjunctival injection, myalgias, and arthralgias. Seven days before onset of symptoms, he had removed a tick from his back while camping in the Paraná Delta in the province of Buenos Aires (Figure 1, panel B), and a painless black eschar appeared subsequently at the bite site. Three days later, he visited a community clinic and received treatment (cephalexin, 500 mg every 6 hours) for presumed cellulitis, without improvement. The patient did not appear severely ill on physical examination. He had a generalized, nonpruritic maculopapular rash that involved predominantly his trunk and extremities but not his face, palms, or soles. Hepatomegaly or splenomegaly, and nuchal rigidity were not found. Laboratory tests showed a serum aspartate aminotransferase level of 67 U/L (reference range <31 U/L), and alkaline phosphatase level of 270 U/L (reference range 40–129 U/L). All other hematologic and biochemical results were within reference ranges. A serum sample and an eschar biopsy specimen were obtained, and the patient was treated empirically with doxycycline (200 mg/d for 6 days). His fever disappeared within 48 hours after starting treatment, and complete remission of symptoms occurred within 6 days.

## Discussion

We confirmed infection with *R. parkeri* in 2 patients bitten by ticks in the Paraná Delta region of Argentina and eschar-associated rickettsioses in 7 other patients from this region and other areas within Argentina (Figure 1, panel B). Because awareness of *R. parkeri* rickettsiosis by clinical practitioners in Argentina is low, and because it appears to be a self-limiting infection, it is likely that many, if not most, cases remain undiagnosed. All case-patients we studied had relatively mild illnesses similar to cases of *R. parkeri* rickettsiosis described in the United States. Indeed, the frequency and character of the clinical features of this disease among case-patients in Argentina closely resemble the signs and symptoms documented in patients in the United States (Table), including the occurrence of an eschar, a maculopapular rash often associated with pustules or vesicles, and the infrequency of gastrointestinal manifestations.

**Table:** Comparison of selected clinical characteristics reported for suspected and confirmed cases of *Rickettsia parkeri* rickettsiosis in patients from Argentina and the United States, 2004–2009*

Clinical characteristic	% Argentina patients, n = 10	% United States patients, n = 15
Fever	100	100
Inoculation eschar	90	93
Rash		
Any type	100	87
Macules or papules	90	87
Vesicles or pustules	50	40
Petechiae	20	13
On palms or soles	20	43
Headache	90	80
Myalgias	70	80
Sore throat	30	NR†
Injected conjunctivae	10	NR
Lymphadenopathy	10	27
Diarrhea	10	0
Nausea or vomiting	0	7
Hospitalization	0	33
Death	0	0

*See (*3*,*5*,*11*). †NR, not reported.

For several years, investigators suspected *R. parkeri* as a cause of at least some of the eschar-associated rickettsioses described in patients from several countries of South America (*5**,**7**–**10*). Our identification of *R. parkeri* DNA in cutaneous lesions of 2 persons in Argentina provides definitive evidence for this *Rickettsia* species as a cause of disease on this continent. During a previous investigation, *R. parkeri* was detected in ≈8% of questing adult *A. triste* ticks collected in the lower Paraná Delta, near the city of Campana (Figure 1, panel B) (*10*). No ticks were saved by patients described in the present report for species identification; however, most patients were bitten while at work or during leisure time in the Paraná Delta during August–November when adult *A. triste* ticks are relatively abundant and actively seeking hosts (*10*). Three cases originated far beyond the boundaries of the Paraná Delta, which suggests that infections with *R. parkeri* might occur in other regions of Argentina. It is also possible that other tick vectors may be involved in the transmission of *R. parkeri* or that other *Rickettsia* species are responsible for these distant cases. Tick- and flea-borne SFG *Rickettsia* spp. identified recently in patients in Argentina and Brazil include *R. felis*, *R.* *massiliae*, and a *Rickettsia* sp. related closely to, but distinct from, *R. parkeri* (*17**–**20*).

Because the patients in this series typically had fever, rash, and myalgias, a diagnosis of leptospirosis or dengue was often considered during initial evaluation. These diseases are endemic across many provinces of northern Argentina, and some cases of *R. parkeri* rickettsiosis are likely to be misdiagnosed as dengue, leptospirosis, or other infectious diseases. Investigations in Mexico and Colombia have identified SFG rickettsioses as common causes of fever among patients believed initially to be infected with dengue virus (*15**,**16*). RMSF, endemic in the province of Jujuy (Figure 1, panel B) and possibly other areas of Argentina, shares several clinical features with *R. parkeri* rickettsiosis. However, it is characteristically a more severe infection that may result in death. By comparison, 6 of 10 persons with confirmed or probable RMSF in Jujuy during 1993–2004 died from this infection, and the surviving patients were hospitalized (*21**,**22*). However, no deaths or hospitalizations were identified among the patients with *R. parkeri* rickettsiosis reported here. Serologic and immunohistochemical tests cannot readily distinguish between these diseases because of cross-reactive epitopes; molecular methods are necessary to identify the specific etiologic agent.

The leading edge of the Paraná Delta is advancing steadily from the deposition of sediments and is expected to reach or surpass the boundaries of the city of Buenos Aires within the next 100 years (*23*). The long-term effects of this process on the distribution and frequency of *R. parkeri* rickettsiosis are difficult to predict; however, as humans continue to encroach on the Paraná Delta through urbanization, agriculture, and tourism, recognized cases of this disease are likely to escalate.
